# Pre-Ramadan health seeking behavior, fasting trends, eating pattern and sleep cycle in pregnant women at a tertiary care institution of Pakistan

**DOI:** 10.12669/pjms.346.15883

**Published:** 2018

**Authors:** Shabeen Naz Masood, Saira Saeed, Nusrat Lakho, Yasir Masood, Muhammad Yakoob Ahmedani, A. Samad Shera

**Affiliations:** 1 *Prof. Shabeen Naz Masood, MBBS, MCPS, FICS, FCPS, PhD, DCPS (HCSM). Department of Obstetrics & Gynecology, ISRA University, Karachi, Pakistan*; 2 *Dr. Saira Saeed, MBBS, FCPS. Department of Obstetrics & Gynecology, ISRA University, Karachi, Pakistan*; 3 *Dr. Nusrat Lakho, MBBS, FCPS. Department of Obstetrics & Gynecology, ISRA University, Karachi, Pakistan*; 4 *Dr. Yasir Masood, M.D. International Centre for Clinical Research Pakistan, (ICCRP), Karachi, Pakistan*; 5 *Prof. M. Yakoob Ahmedani, FCPS. Department of Medicine, Baqai Institute of Diabetology and Endocrinology, Baqai Medical University, Karachi, Pakistan*; 6 *Prof. A. Samad Shera, FRCP. Diabetic Association of Pakistan, WHO Collaborating Centre, Karachi-Pakistan*

**Keywords:** Ramadan Fasting in Pregnancy, Sleep Cycle, Eating Pattern

## Abstract

**Objective:**

To observe the pre-Ramadan health seeking behavior, fasting trends, eating pattern and, sleep cycle in pregnant women.

**Methods:**

It is a cross-sectional observational study, from July to September 2017, conducted at Tertiary Care Hospital in Karachi. The tool used for data collection was interviewer based closed ended questionnaire, 279 pregnant women who fasted during Ramadan were included in the study.

**Results:**

One to ten days of fasting was observed by 85.7% (198) of women. About 72.4% (202) never consulted any doctor for pre-Ramadan advice regarding fasting in pregnancy. Pregnant women 81.7% (228) believed that fasting would not cause any harm to their unborn child, while 42.7% (119) of family members feared about the health of mother and unborn child. Seventy four percent (208) of respondents had a reduced sleep cycle of about 3-4 hours. The food items consumed at Sehri and Iftar were rich in carbohydrates and fats.

**Conclusions:**

Pre-Ramadan medical consultation regarding safety of fasting during pregnancy should be structured and customized for women and their families. Gaps in knowledge identified in this study may help healthcare professionals to address these issues.

## INTRODUCTION

Ramadan is in the 9^th^ lunar month of Islamic calendar in which fasting is made obligatory on Muslim adults; where they are required to refrain from food, beverages, oral drugs, medications and smoking between dawn and sunset.^1^,^2^ Duration of Ramadan fasting (RF) is variable globally according to geographic and seasonal variations from 12 to 19 hours.^3^

Limitation of fluid intake during fast, especially in summers, is likely to cause dehydration, hypotension, hypovolemia, increase blood viscosity,^4^-^6^ and risk of preterm labour if the fast is observed before 20 weeks.^7^

Exemption from fast is allowed in Holy Quran for people with illness and travellers. The QURAN verses regarding fasting are: “You who believe, fasting is prescribed for you, as it was prescribed for those before you, so that you may be mindful of God, and anyone who is ill or on a journey should make up for the lost days by fasting on other days later”.^8^

According to clear rulings in Hadith the exemption from fast is permissible to pregnant woman if she fears harm to her health or the health of her unborn child.^9^-^12^

In spite of exemptions from fasting during pregnancy, in most of the Muslim cultures and ethnicities woman do not wish to forgo fasting. The clinicians are often confronted with the dilemma whether to advise fast or not during pregnancy. The caring physicians should approach this issue with cultural sensitivity. They should be well informed about the religious rulings, references and guidelines about fasting during pregnancy and clinical management of fasting related dysglycaemia. It was a cross-sectional observational study, the aim of study was to observe the pre-Ramadan health seeking behavior, fasting trends, eating pattern and sleep cycle in pregnant women.

## METHODS

The duration of study was from July to Sept 2017, at Gynaecology Department of ISRA University Karachi Campus. Informed consent was taken from study participants and the tool used for data collection was interviewer based closed ended questionnaire. The sample size was calculated from the online software raosoft.com by taking 95% CI and 5% of margin of error required for sample of 279. Ethical approval was taken from hospital ethics committee. Pregnant women who ever fasted during Ramadan were included in the study.

## RESULTS

A total of 279 pregnant women were enrolled in this study. Mean age of respondents was 25.9±5.1 with the range of 16 to 40 years. The education level was below 10^th^ grade in 59.50% (166) and 98% of the respondents were housewives. The most spoken languages were Balochi 32.97% (92), Sindhi 31.90% (89), and Urdu 26.88% (75) (Table-I). Majority of the women 63.4% (177) were primigravida. A large number of women 70% (195) attended OPD for the first time in third trimester of pregnancy. Although majority of pregnant women 82.8% (231) fasted during the month of Ramadan, 85.7% (198) fasted only for 1-10 days. The women were interviewed at their antenatal visit in Ramadan, irrespective of period of gestation. Mean fasting days were 5.55 with standard deviation of 7.96.

**Table-I:** Demographic Features of study population (n = 279).

Age Group	Frequency	Percent
16-25 years	156	55.9%
26-35 years	112	40.1%
36-45 years	11	3.9%
***Education***		
Primary education	166	59.50%
Matric	61	21.86%
Intermediate	39	13.98%
Graduate	13	4.66%
***Profession***		
Religious scholar	1	0.35%
Housewife	274	98.2%
Nurse	2	0.71%
Tailor	1	0.35%
Teacher	1	0.35%
***Language***		
Balochi	92	32.97%
Sindhi	89	31.90%
Siraiki	2	0.72%
Urdu	75	26.88%
Punjabi	9	3.23%
Pashto	12	4.30%

Feeling of weakness was the prime reason to skip the fast in 81.2% (39) and for similar reason 58% (162) for women considered Ramadan Fasting (RF) difficult during pregnancy and 6.1% (17) woman broke the fast due to weakness (Table-II). Regarding the religious knowledge, 95.7% (265) of the women said that fasting was compulsory (farz) during pregnancy, and 87-95% (244-265) of respondents said that during pregnancy & lactation women should observe RF. Fifty percent (139) of women said that if they were not able to fast during Ramadan they could redo it while 37.6% (105) said that they would give fidya (compensation) for not fasting (Table-III).

**Table-II:** Fasting Trends (n = 279).

Fasting Trends	Frequency	Percent
Primary gravid	177	63.4%
Multi gravid	102	36.5%
***Period of Gestation***		
1^st^ Trimester	11	3.9%
2^nd^ Trimester	73	26.2%
3^rd^ Trimester	195	69.9%
***Fasting during pregnancy***		
No	48	17.2%
Yes	231	82.8%
***Number of days of women fasted***		
1-10 days	198	85.7%
11-20 days	11	4.8%
21-30 days	22	9.5%
***Reason for not fasting (n = 48)***		
Feeling of weakness	39	81.2%
The unborn child may be under-weight	1	2.1%
Family members advising not to fast	2	4.2
Health care providers advising not to fast	6	12.5%
***Pre-Ramadan Medical Consultation for fasting***		
No	202	72.4%
Yes	77	27.6%
***If yes, by whom (n=77)***		
We know it better	9	11%
Doctor	10	12.9%
Nurse	7	9.09%
Muslim Scholars	23	29.8%
Family Members	28	36.3%
***Reason for not consulting in pre-Ramadan period (n=202)***		
Unaware of its importance	185	91.5%
Doctor will not allow for fasting	17	8.5%
***Did Doctor Allow to fast (n=77)***		
No	24	31.2%
Yes	53	68.8%

**Table-III:** Beliefs regarding fasting (n = 279)

Beliefs regarding fasting in pregnancy	Frequency	Percent
Farz (Compulsory)	265	95.7%
Sunnah	4	1.4%
Fasting is not necessary. (If we fast it will give reward. If do not fast then there is no Gunnah (Punishment)	8	2.9%
***Mother who breastfeed, should they fast?***		
No	30	10.8%
Yes	244	87.5%
Don’t Know	5	1.8%
***Do you think that pregnant women should fast during Ramadan?***		
No	14	5.0%
Yes	265	95.0%
***Why it is harmful to fast during pregnancy?***		
Your health	7	2.5%
Health of your unborn child	22	7.9%
Harmful to both of you	21	7.5%
It will not cause any harm	228	81.7%
Any other	1	0.35%
***Is fasting during pregnancy difficult?***		
No	117	41.9%
Yes	162	58.1%
***Why it is difficult? (n=162)***		
You feel very weak	60	37.0%
Feel more hungry	51	31.5%
Feel more thirsty	37	22.8%
Feel more sleepy	14	8.6%
***Fears of spouse about fasting in pregnancy***		
She will be weak	154	55.2%
Fear of harm to health of fetus	6	2.2%
The health of mother and unborn child could be effected	119	42.7%
***Do fetal movement decrease during fasting?***		
No	97	34.8%
Yes	99	35.5%
Don’t know	83	29.7%

About pre-Ramadan medical consultation regarding RF in pregnancy, 72.4% (202) of women did not consult any doctor for this particular reason, 91.5% (185) were not aware about its importance while 8.5% (17) feared that they may not be allowed to fast if consulted (Table-II).

Regarding perception about harm to fetus or unborn child, 81.7% (228) of pregnant women thought that fasting would not cause any harm to either of them. However, 42.7% (119) of family members and spouse did have apprehensions about the health of mother and unborn child.

**Fig.1:**
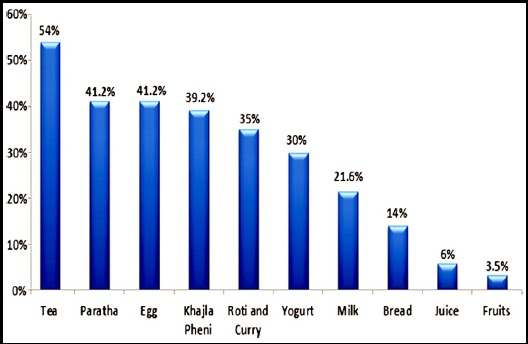
Common food items consumed at Sehri n=231.

**Fig.2:**
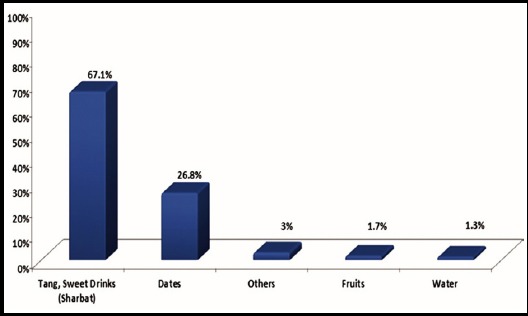
Food items consumed for opening the fast n=231.

**Fig.3:**
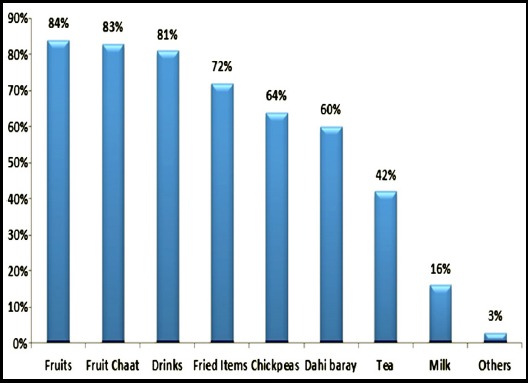
Dietary items commonly consumed at Iftar meal n=231.

No significant difference was perceived in fetal movements during fasting and after iftar in 29.7% (83) of pregnant women (Table-III). Regarding sleep pattern 74% (208) of respondents slept only for 3-4 hours during Ramadan, 14% of respondents slept for 5-6 hours and only 12% of participants slept for >7 hours. The food items most commonly consumed at Sehri were khajla pheni 42% (96), omlet 40% (93), paratha 39% (90). Tea was most commonly consumed beverage by 54% (124) of participants (Fig.1). Sixty seven percent (155) of women preferred to open the fast with sweet drinks (sharbat) while 27% (62) of women used dates (Fig.2), however food items commonly consumed for iftar dinner were fruit chaat 83% (192), fried items (samosa, tempura) 72% (167), chick peas 64% (148) and dahi baray 60% (139) (Fig.3).

## DISCUSSION

A large number of pregnant women preferred to observe Ramadan fast in spite of religious exemptions given to them during pregnancy and lactation.^9^-^12^ There is conflicting literature about safety of RF during pregnancy. The available literature is scare about women’s perceptions and beliefs regarding RF. The health care professionals are unequipped with appropriate insights, knowledge and proper counseling about the safety and best practices of RF during pregnancy.

In our study a large number of women 82.8% did fast during Ramadan, however most of them 86% fasted only for 1-10 days. It was observed from the study that difficulty to fast, for a larger number of days was due to feeling of weakness in pregnancy. This finding is similar to a study conducted by Sultan IE et al whereby 50% of the women quit fast due to similar reason while in another study 51.2% participants quit fasting for no particular reason.^13^,^14^

Parity has a comparable effect on RF as 63.4% of study subjects were primigravidas, while studies conducted in Iran and Singapore show women with higher parity who observed fast.^13^,^15^

In our study 72.4% of pregnant women did not seek a formal pre-Ramadan consultation regarding safety of RF though they did attend antenatal clinics. The main reason for non-consultation was unawareness about its importance in 91% of respondents. A large number 29.8% of pregnant women consulted Muslim scholars about safety of RF which is significantly higher than the practices seen in USA (19%).^16^ One interesting observation about Ramadan specific consultation, whereby 11% of the pregnant women thought that they knew it better to decide to fast or otherwise. This is a message to healthcare professionals that they should not only acquire adequate knowledge about Islamic rules regarding safety of fasting but must also possess appropriate skills to communicate and customize counseling according to people’s beliefs and practices about RF. In Robinson’s study only 3% of pregnant women said that they were their own doctors and they themselves could decide whether to fast or not.^17^ Whereas in our study 13% of women placed their trust on healthcare professionals and agreed to follow their advice. In spite of this trust on healthcare professionals 8.5% of them had apprehension that they may not be allowed to fast if consulted. These perceptions about healthcare professionals were also portrayed in another study whereby 17% of women apprehended that doctors were too judgmental and disrespectful regarding advice for fasting in pregnancy.^17^

In our study 36.3% of women placed their trust on family members for decision to fast. One of the studies show higher support (90%) given by family members in deciding women to fast during pregnancy.^14^ Therefore family members must be included in pre-Ramadan consultation to help pregnant women to observe religious duties, provided it is safe for fetal and maternal health.

Most of the pregnant women (95.7%) possessed adequate knowledge about Islamic principles of Ramadan fasting, but were unaware about the religious exemptions provided to them that they could avoid fast during pregnancy and lactation. Whereas in Mubeen’s study,^18^ a higher number of women (54%) had formal education, and 88% of them considered fasting as an essential duty.

A large number of study participants 81.7% believed that fasting would not cause any harm to their unborn child, and various studies concluded Ramadan fasting did not have any effect on fetal and neonatal birth indices, like weight, height, head circumference, apgar score and children’s IQ.^7^,^19^,^20^ While a study reported by Khoshdel A shows increased rate of preterm labour and growth restriction in fasting mothers, however this study has many methodological flaws, it is a self-control cohort study with a very small sample size (39) and short duration.^21^

Although maternal perception of fetal movements (FM) is very subjective; no significant difference in FM was noted during fasting and after iftar. Similarly one of the studies also inferred that 97% of women perceived no effect on fetal movements, no changes in fetal Cardiotocography and Doppler flow velocities during fasting.^22^,^23^

When women were asked about sleep deprivation during Ramadan, 74.6% of them said that their sleep time was reduced to 3-4 hrs/day due to altered sleep wake cycle. They woke up early in the morning to prepare Sehri meals for the family. After morning prayers, sent children to school and later on took care of home chores and had busy afternoons in preparation of meals for iftari. Generally this chain of events leave women sleep and rest deprived. This may lead to many maternal effects such as prolonged labour, increased intensity of labour pains, increased rate of cesarean section, and postpartum depression. Maternal sleep deprivation may lead to preterm birth and prematurity is known to be a leading cause of perinatal mortality and morbidity.^24^

During the month of Ramadan people from varied sociocultural backgrounds have different dietary preferences mostly comprising of high carbohydrates and fats.^25^ The literature search did not mention the names of specific food items consumed by the people during Ramadan. However, in our study common sehri food items consumed were tea, paratha, omelet and khajla pheni. Whereas at iftari sweet drinks (sharbat) were the most commonly consumed beverages for opening the fast while dates were used by only 26% of participants. This is followed by iftari meal which mostly consists of fruits, fried items (tempura, samosa, chana) drinks and dahi baray. These edibles are high in carbohydrates and fats, this may lead to significant rise in cholesterol levels in Pakistani population because of their dietary habits.^3^ Almost 42% of participants consumed tea at sehri, which could increase thirst and urination that may lead to disturbances in fluid balance in pregnant women in hot and humid climate. Only 3-6% of our study participants consumed juices and fruits while the dietary recommendation is to consume ample amount of fruits in pregnancy.

### Limitations

CTG is an objective fetal surveillance tool, to confirm the subjective evidence of less fetal movements. This tool was not used in this study. We noted the type of food consumed but did not quantify the caloric count, lipid and cholesterol levels. This is a cross sectional study and is not generalizable. The estimation of sleep duration was subjective in this study.

## CONCLUSION

Pre-Ramadan medical consultation should be structured and customized according to faith and beliefs of women and their families. Appropriate dietary advice, importance of adequate sleep along with readjustment of medications can reduce the maternal and fetal health risks. This will be a good addition to existing Ramadan specific guidelines for health care professionals. Pregnant women and family members should be involved in decision making and their apprehensions should be addressed regarding safety of fasting during pregnancy in pre-Ramadan period.
